# Factors affecting the quality and nutritional value of donkey meat: a comprehensive review

**DOI:** 10.3389/fvets.2024.1460859

**Published:** 2024-09-06

**Authors:** Wei Zhang, Min Zhang, Yujiang Sun, Shuqin Liu

**Affiliations:** ^1^College of Animal Science and Technology, Qingdao Agricultural University, Qingdao, China; ^2^Shandong Provincial Animal Husbandry Station, Jinan, China; ^3^Gene Bank of Equine Genetic Resources, Qingdao, China

**Keywords:** donkey, meat quality, nutritional value, breed, feeding regimen, muscle type

## Abstract

Donkey meat is characterized by a high content of proteins, essential amino acids, and unsaturated fatty acids and is low in fat, cholesterol, and calories. Thus, it is considered a high-quality source of meat. Based on the data from PubMed and Web of science within past 10 years, this review summarizes the factors affecting the quality of donkey meat and its nutritional value, including breed, genetics, gender, age, muscle type, feeding regimen, storage and processing conditions. Breed, gender, age, and feeding regimen mainly affect the quality of donkey meat by influencing its intramuscular fat content and carcass quality. Meanwhile, the tenderness and flavor of donkey meat depend on the muscle type, storage and processing conditions. Genetics, on the other hand, fundamentally affect donkey meat quality by influencing the polymorphism of genes. These findings provide valuable insights and guidance for producers, consumers, and decision-makers in the donkey meat industry, promoting the development of more effective marketing strategies and the improvement of meat quality, thereby enabling the expansion and progress of the entire industry.

## 1 Introduction

Donkeys have historically played a crucial role in agricultural production. However, as production processes, machinery, and lifestyles have evolved, donkeys are now increasingly being used for the production of meat, skin, dairy, and other products. In particular, donkey meat production has emerged as an important area of focus. From an economic perspective, China boasts one of the largest populations of donkeys around the world. This has promoted the development of a significant industry centered around donkey meat, milk, and hide production in rural areas of China. Notably, this industry has become a crucial source of income for local people living in rural areas, stimulating the local economy (1, 2).

From a nutritional perspective, donkey meat may have a better nutritional value than pork and mutton. Donkey meat is high in protein and low in fat. A study has shown that a high-protein diet may lower liver fat by inhibiting fat absorption and reducing fatty acid biosynthesis, so donkey meat may be an excellent choice for individuals pursuing fitness goals or aiming to lose weight (3). Furthermore, promoting the growth of the donkey meat industry can enhance the diversity of meat production in China. Currently, livestock such as cattle, pigs, and sheep dominate the meat market (4, 5). However, introducing donkey meat as a new option could expand consumers’ choices, thus decreasing the over-reliance on traditional livestock. Notably, donkeys not only possess remarkable adaptability and resilience to drought and cold but also thrive in harsh environmental conditions (6–8). In certain environments, donkeys may prove more cost-effective to raise and may utilize resources more efficiently than other livestock, thereby reducing the pressure on environmental resources. In essence, expanding the donkey meat industry could have several benefits — diversifying meat production, reducing dependence on traditional livestock, and enhancing resource efficiency.

Nonetheless, the donkey meat industry in China is still in its nascent stages due to the lack of foundational research and the dearth of standardized feeding practices. To aid in the advancement of the donkey industry, this paper comprehensively reviews the primary factors known to influence donkey meat quality. We believe this review could provide the latest progress of producing high-quality meat and new insights for product innovation in the donkey meat industry.

## 2 Nutritional value of donkey meat

Donkey meat has a high nutritional value and unique flavor. Compared with other popular types of meat such as beef and mutton, donkey meat has a higher content of proteins, vitamins, and minerals and is lower in fat and cholesterol (Table 1) (9–11). Thus, from a health perspective, donkey meat is a good alternative to traditional meats (12).

**Table 1:** Nutritional composition of meat (84, 85).

Nutrients	Donkey meat	Chicken meat	Beef	Pork
Protein (g/100 g)	22.8	24.1	20.3	20.8
Fat (g/100 g)	3.5	8.9	8.1	13.2
Vitamin B12 (μg/100 g)	1.9	0.37	2	1
Na (mg/100 g)	36.8–83.6	72	60	53
P (mg/100 g)	185–335	200	145	221
Fe (mg/100 g)	2.86–4.77	1	1.5	0.6
Zn (mg/100 g)	2.99–4.71	0.8	3.6	1.6

Intramuscular fat (IMF) content is an important determinant of meat quality characteristics such as tenderness and juiciness (13, 14). Thus, it is an important index for measuring meat quality (15). Meat with a low IMF content is drier and has a poor flavor. Intracellular fatty acids, which can act individually or form larger molecules, have diverse functions (16). Donkey meat has a higher content of essential fatty acids, especially polyunsaturated fatty acids (PUFA), than livestock and poultry meats (17, 18). The high ratio between unsaturated and saturated fatty acids (SFA) in donkey meat induces a high degree of unsaturation in the IMF of donkey meat (19, 20). Additionally, the content of essential amino acids (essential AA) in donkey meat is higher than that of non-essential amino acids (non-essential AA) (21).

The most common nutritional indices of donkey meat, including the index of atherogenicity (IA) and thrombogenicity (IT) (22) and the health promotion index (HPI) (23), have been estimated using the below-mentioned formulae (24) (Table 2).


$$IA=\left[C12:0+\left(4\times C14:0\right)+C16:0\right]/\left(\sum UFA\right)$$

$$\begin{array}{l} \mathrm{IT}=\left(C14:0+C16:0+C18:0\right)\\ /\left[\begin{array}{l} \left(0.5\times \sum \mathrm{MUFA}\right)+\left(0.5\times \sum n6-\mathrm{PUFA}\right)\\ \;+\left(3\times \sum n3-\mathrm{PUFA}\right)+\left(n3/n6\right) \end{array}\right] \end{array}$$

$$HPI=\left(\sum UFA\right)/\left[C12:0+\left(4\times C14:0\right)+C16:0\right]$$

**Table 2:** Nutritional value assessment of donkey meat (9).

Nutritional index	Donkey meat
IA (g/100 g)	0.55
IT (g/100 g)	0.81
HPI (g/100 g)	1.81

IA, index of atherogenicity; IT, index of thrombogenicity; HPI, health promotion index.

Flavor is a sensory attribute of food that is perceived through its taste and smell and is the main determinant of meat quality (25, 26). Aldehydes are important flavor compounds in the meat derived from donkeys, cattle, pigs, and sheep. Among these different types of meat, donkey meat has the highest content of aldehydes. Specifically, aldehydes — especially hexanal — account for 76.39% of the total flavor compounds present in donkey meat (27). Moreover, owing to its high content of sweet and umami amino acids, donkey meat is more flavorful than other meats. Thus, it has the potential to become a unique and preferred choice of meat for a wide range of consumers.

## 3 Effect of breed on donkey meat quality

The emergence of donkey breeds, uniquely adapted to the specific characteristics of their native regions, was influenced not only by geographical and climatic conditions but also by human-selected breeding (28–31). A comparison of carcass traits and meat quality between two donkey breeds from the Mediterranean region showed that North African donkey (NAD) meat had a higher final weight, IMF content, and hue value, while Masri donkey (MD) meat had a higher cold dressing percentage and cooking loss value (21). Given that high cooking loss values can reduce meat tenderness, these findings indicated that NAD meat is more tender.

As mentioned previously, the content of fatty acid plays a crucial role in determining meat quality. One study compared the fatty acid content of meat from Burguete foals (BF) and Hispano-Breton foals (HBF). This study found that the content of SFA and mono-unsaturated fatty acids (MUFA) was lower in the meat from BF than in that from HBF, while the content of PUFA was higher (32).

Interestingly, the quality of donkey meat can differ even among different subpopulations of the same breed. A total of 38 volatile compounds (VOCs) were identified in the meat from SanFen and WuTou donkeys, two lines of Dezhou donkeys. Notably, the ketone and alcohol content of SanFen donkey meat was significantly higher than that of WuTou donkey meat, while the content of aldehydes showed the opposite trend (33).

In summary, the quality of meat from different donkey breeds differs due to variations in carcass quality, fatty acid content, flavor compound levels, and other factors (Table 3). Therefore, cross-breeding can be used to combine excellent meat traits and thus improve the quality of donkey meat.

**Table 3:** Comparison of quality parameters between different varieties of donkey meat (21, 32, 33).

Meat quality indicators	MD	NAD	BF	HBF	SanFen	WuTou
Cooking loss (%)	43.92 ± 0.82^a^	39.95 ± 1.23^b^	-	-	-	-
IMF (%)	0.98 ± 0.13^b^	4.49 ± 0.73^a^	2.08 ± 0.106	2.22 ± 0.145	-	-
PUFA (%)	26.41 ± 2.61	28.04 ± 2.70	20.45 ± 0.833^b^	24.65 ± 0.549^a^	-	-
MUFA (%)	34.63 ± 3.30	34.28 ± 3.32	41.09 ± 0.596^a^	39.53 ± 0.556^b^	-	-
Flavor profile	-	-	-	-	High in ketones and alcohols	High in aldehydes

Different letters (a, b) indicate significant differences at *p* < 0.05. MD, Masri donkey; NAD, North African donkey; BF, Burguete foals; HBF, Hispano-Breton foals. IMF, intramuscular fat; PUFA, polyunsaturated fatty acids; MUFA, mono-unsaturated fatty acids (the same below).

## 4 Effect of genetics on donkey meat quality

Gene regulation plays a key role in determining meat quality, and genetics can lead to variations in meat quality traits and the quality of meat products (34–37). The *LTBP2* gene polymorphism is significantly correlated with the body size and carcass traits of Dezhou donkeys and has potential applications as a molecular marker to improve the production performance of donkeys (38). *HOXC8* is an important gene affecting the number of multiple vertebrae and carcass weight in Dezhou donkeys, and g.15179224C>T and g.15179674G>A may be potential genetic markers for screening and breeding new high-quality and high-yield donkey varieties (39). In a study investigating the relationship between the *NKX 1–2* gene and donkey meat quality, *NKX 1–2* was identified as a candidate gene for breeding Dezhou donkeys for meat, and the g.54704925 A>G locus emerged as a marker locus for the selection and breeding of good-quality animals (40). These findings show that gene polymorphisms can affect the quality of donkey meat.

Omics technology, including transcriptomics, proteomic, genomic, and metabolomics, is an important tool for studying the biological characteristics of living organisms (41, 42). In a transcriptomic study of donkey meat, RNA sequencing (RNA-seq) was employed to identify the differentially expressed genes (DEGs) between animals with a high and low IMF content in the *longissimus dorsi* muscle (LDM). In total, 887 genes were identified, of which 167 DEGs were enriched in multiple biological processes and pathways related to adipocyte differentiation, lipid synthesis, and neutral lipid metabolism (43). A study using RNA-seq to identify important genes in Dezhou donkey muscles revealed that the DEGs were mainly involved in bioregulatory and metabolic processes in *gluteus* and *longissimus dorsi* meat. Additionally, variations in meat tenderness were related to muscle fiber type and glucose metabolism, which were linked to DEGs such as *MYH1*, *MYH7*, *TNNC1*, *TNNI3*, *TPM3*, *ALDOA*, *ENO3*, and *PGK1* (44). Meanwhile, a proteomic investigation into donkey meat traits revealed that the most important pathways contributing to meat quality differences between the *semitendinosus* (ST) and the *longissimus thoracis* (LT) muscles were the GnRH and MAPK pathways. Meanwhile, the pathways of fat digestion and absorption and the regulation of the actin cytoskeleton were found to affect differences in meat quality between the *gluteus maximus* (GM) and LT muscles (45). A lipidomics and volatomics study of donkey meat found that as the IMF content of donkey meat increased, the content of MUFA (especially oleic acid) also increased significantly, while that of SFA decreased significantly (46).

Increased genetic diversity within breeds may lead to improvements in the quality and yield of donkey meat, as more genetic variation offers more options for breeding individual animals with superior meat quality. With inter-breed genetic diversity, new genetic information can be introduced through mating to potentially improve meat quality and prevent the accumulation of genetic defects. In general, genetic diversity is a significant factor affecting the quality of donkey meat. Through scientific breeding and management measures, genetic diversity can be leveraged to improve the quality and yield of donkey meat and meet the demand for high-quality donkey meat. However, the genetic regulation of meat quality is complicated. Most meat quality traits are quantitative traits and are controlled by multiple genes. Although several candidate genes related to the quality of donkey meat have been screened via multi-omics methods, the molecular mechanisms governing meat quality warrant further elucidation.

## 5 Effect of gender on donkey meat quality

The effect of sex on the quality of donkey meat is not obvious and is primarily mediated through sex-related differences in carcass weight. One study investigated the effects of sex on the weight, physicochemical properties, nutritional value, and sensory characteristics of meat from 15-month-old donkeys. This study found that sex had a significant effect on the fatty acid content of the longissimus dorsi. Additionally, the slaughter weight in the male group was slightly higher than that in the female group. However, no significant sex-related differences in carcass measurements, meat chemical composition, meat color, meat texture, amino acid content, and sensory characteristics were observed (47). These results were consistent with reports on meat quality in “Galician Mountain” foals (GMF) (48). Among Catalan crossbreed donkeys, males were found to have a higher carcass weight and subcutaneous fat content than females (49). Collectively, these studies show that sex affects the fat content of donkey meat, with male donkeys having a slightly higher overall fat content than female donkeys.

## 6 Effect of age on donkey meat quality

Age can significantly affect the quality of donkey meat. In one study on foals who were naturally suckled on maternal milk (NS), the foals aged 12 months and 18 months showed differences in the moisture, protein, IMF, and fatty acid content of meat (50). In Martina Franca (MF) donkeys, 12-month-old foals were found to have a higher carcass weight, dressing percentage, and muscle glycogen content than 8-month-old foals. Meanwhile, the meat from 8-month-old donkeys showed lower shear force values (51). Another study on GMF revealed significant differences in carcass weight, meat quality (chemical composition, color characteristics, and texture characteristics), and nutritional value (fatty acid and amino acid composition) between the meat from 8- and 11-month-old foals. Notably, the carcass weight of foals increased with age. However, meat from 8-month-old foals had higher cholesterol and color luminosity values than meat from 11-month-old foals. In terms of fatty acid content, 8-month-old foals had the highest n-3 fatty acid content (10.99%) and the lowest n-6 fatty acid content (11.76%) (52).

To further understand the effects of age on donkey meat quality, we integrated the results of these three previous studies (Table 4). Accordingly, we inferred that as the age of donkeys increases, the moisture content of donkey meat gradually decreases while the protein and IMF content gradually increases. This is consistent with the age-related changes in the quality of pork and beef (53, 54). Additionally, the content of essential AA is higher than that of non-essential AA in donkey meat, although the contents of SFA, MUFA, and PUFA appear to be comparable across different ages. Importantly, as the age increases, the shear force of donkey meat increases, thereby increasing its toughness.

**Table 4:** Effects of age on quality of donkey meat (50–52).

Parameter	NS		MF		GMF	
Age (month)	12	18	8	12	8	11
Moisture (%)	75.44^a^	72.64^b^	77.3	75.8	74.54	74.78
Protein (%)	19.83^a^	21.35^b^	19.8	21.0	20.41	21.05
IMF (%)	1.13^b^	1.94^a^	1.76	1.87	1.27	1.29
Shear force (N)	-	-	54.03^b^	60.66^a^	-	-
Essential AA (%)	-	-	9.36	10.05	9.53	9.40
Non-essential AA (%)	-	-	8.37	9.55	8.97	8.84
SFA (%)	43.40^a^	46.88^b^	40.15	39.97	37.66	36.45
PUFA (%)	19.84^b^	19.26^a^	26.36	26.43	22.81	22.75
MUFA (%)	35.05	36.15	33.20	33.45	39.53	40.79

Different letters (a, b) indicate significant differences at *p* < 0.05. NS, donkey in naturally suckled on maternal milk; MF, Martina Franca donkey; GMF, “Galician Mountain” foals. Essential AA, essential amino acids; Non-essential AA, non-essential amino acids; SFA, saturated fatty acids (the same below).

## 7 Effect of muscle type on donkey meat quality

In donkeys, different muscles exhibit great differences in fatty acid, IMF, and collagen content. In a study on Dezhou donkeys, the IMF content of the LDM was found to be higher than that of the *rump* muscle (RM) and *hamstring* muscle group (HM). Notably, triglycerides (TG) rich in SFA and MUFA accounted for a majority of the IMF in the meat from these donkeys (55). Polidori and Vincenzetti (56) slaughtered 10 entire crossbred donkeys (EC) and found that the content of fat and PUFA in the *semimembranosus* (SM) muscle was significantly higher than that in the ST muscle, while the collagen content was significantly lower in the SM muscle.

Similarly, differences in VOCs content and tenderness have also been observed among different muscles. Previously, headspace-gas chromatography-ion migration spectrometry combined with stoichiometric analysis was used to study the VOCs in the LDM, GM, and *biceps femoris* (BF) muscles of Dezhou SanFen donkeys. The findings showed that the levels of benzaldehyde, 2-heptanone, 3-methylbutanal, 2-butanone, and 2-butanone-D were significantly higher in the LD and GM muscles than in the BF muscle, whereas nonanal showed the opposite trend (57). In a study on the physicochemical properties of different cuts of meat from Dezhou black donkeys, the shear force and hardness of LD and BF meat were found to be significantly lower than those of GM meat. Meanwhile, the contents of IMF and MUFA in the LD muscle were significantly higher than those in the GM and BF muscles, while the contents of PUFA showed an inverse trend (58).

Our meta-analysis of these above studies (Table 5) indicated that the IMF content of the LDM was the highest among Dezhou donkeys, suggesting that the LDM of donkeys has a good fat-storage capacity. Compared with other types of muscles, the LDM had a relatively low shear force and hardness, while these indexes were the highest in the GM muscle. This may be because the GM muscle is employed frequently during exercise, increasing the muscle fibers in this muscle. As a result, the shear force of the GM muscle is high, which decreases its tenderness. Notably, the LDM of Dezhou donkeys had the highest content of SFA and MUFA and the lowest content of PUFA, which are easily oxidized (59, 60). Therefore, overall, the LDM appeared to show a high IMF content, tenderness, and antioxidant capacity and was found to be the most suitable part of Dezhou donkey meat for consumption. In EC donkeys, the ST and SM muscles exhibited significant differences in the concentration of fatty acids and collagen, which indicated that there are significant differences in the quality of donkey meat obtained from different muscle types. Therefore, different cuts of donkey meat can be sold and utilized to improve the economic value and use efficiency of donkey meat.

**Table 5:** Quality of donkey meat from different muscles (55–58).

Variety	DZ						EC	
Parts	LDM	RM	HM	LDM	GM	BF	ST	SM
TG (%)	74.09^b^	72.97^ab^	70.29^a^	-	-	-	-	-
SFA (%)	33.5	33.71	33.28	34.26	33.71	33.02	43.82	42.17
MUFA (%)	38.8^c^	29.56^a^	33.2^b^	40.43^b^	30.07^a^	34.26^a^	35.63	35.74
PUFA (%)	22.28^a^	29.7^b^	29.46^b^	23.68^a^	31.95^b^	29.32^b^	20.61^a^	22.11^b^
IMF (%)	3.82^b^	2.37^a^	2.25^a^	2.31^b^	1.27^a^	1.32^a^	-	-
Shear force (kgf)	-	-	-	3.18^a^	3.89^b^	3.06^a^	-	-
Hardness (kgf)	-	-	-	1.40^a^	2.35^c^	1.75^b^	-	-
Collagen concentration (μg/mg)	-	-	-	-	-	-	44.2^a^	32.1^b^
Benzaldehyde	-	-	-	132.62^b^	146.36^b^	49.8^a^	-	-
2-Heptanone	-	-	-	115.02^b^	117.4^b^	93.07^a^	-	-
3-Methylbutanal	-	-	-	124.74^b^	103.52^b^	59.32^a^	-	-
2-Butanone	-	-	-	733^b^	698.21^b^	528.22^a^	-	-
2-Butanone-D	-	-	-	295.93^b^	255.15^b^	116.12^a^	-	-
Nonanal	-	-	-	154.64^a^	148.52^a^	232.53^b^	-	-

Different letters in the same row indicate significant differences (a, b: *p* < 0.05; c: *p* < 0.01). DZ, Dezhou donkey; EC, entire crossbred donkey; LDM, longissimus dorsi muscle; RM, rump muscle; HM, hamstring muscle; GM, gluteus maximus muscle; BF, biceps femoris muscle; ST, semitendinosus muscles; SM, semimembranosus muscle; TG, triglycerides.

## 8 Effect of feeding regimen on donkey meat quality

The nutrients absorbed by animals from the feed are used for their own maintenance, growth, lactation, and other physiological processes. The feeding regimen mainly includes the feeding method and the type of diet. These factors complement each other and have various effects on the quality of donkey meat.

### 8.1 Feeding method

The primary feeding methods for donkeys include centralized feeding in the shed, grazing and free feeding, and suckling. Numerous studies have shown that the feeding method can affect meat quality in pigs, cattle, sheep, chickens, and other animals (61–64). In donkeys, housed feeding and free-range grazing can have varying effects on meat quality. In a previous study, 24 hybrid male donkey foals were reared under an intensive or extensive feeding system. This study showed that the average daily gain, carcass weight, and some meat quality parameters are significantly affected by the feeding system. Additionally, the IMF content, SFA content, and tenderness of donkey meat were higher if the animals were reared under intensive feeding. Meanwhile, the contents of protein and unsaturated fatty acids, including n-3 essential fatty acids, were significantly higher in meat from animals reared under the extensive feeding system (65). In GMF reared under free-extensive and semi-extensive feeding modes, the content of PUFA was significantly higher in the meat derived from the free-extensive production group, while the content of MUFA was significantly higher in the meat derived from the semi-extensive production group (66). These findings suggest that intensive feeding is more appropriate for fattening, while extensive free-range feeding enables the production of lean meat with a higher protein content.

Natural suckling and artificial suckling can also have different effects on the quality of donkey meat. In studying the effects of different suckling methods on the quality of MF donkey meat, De Palo et al. (50) found that meat from artificially suckled donkeys had a higher IMF and protein concentration. Moreover, the meat from naturally suckled donkeys was darker and redder than that from artificially suckled donkeys. Two other studies by De Palo et al. (67, 68) found that artificial suckling regimes could improve the growth rate of donkey foals, especially during the first 6 months of life, resulting in a significant increase in weekly gain, final live weight, and carcass weight. Additionally, the type of suckling also affected lipid peroxidation patterns. These results indicate that from a nutritional perspective, the contents of protein and IMF are better in meat from artificially suckled donkeys. However, in terms of appearance, meat from naturally suckled donkeys appears to be superior.

### 8.2 Diet type

The type of diet mainly affects meat quality by influencing the intake and absorption of nutrients, which are essential for meeting the physiological needs of animals. Several studies have shown that the dietary levels of different nutrients have important effects on meat quality (69–72). For donkeys, the effect of diet on meat quality is mainly through the influence of carcass weight and daily gain. In a study of Dezhou donkeys, the average weight gain, body circumference, and carcass percentage were found to be significantly higher in the 1.50 and 1.25% concentrate feed groups than in the 1.00% concentrate feed group. Based on various basic indicators, this study concluded that 1.25% concentrate feed was optimal for Dezhou donkeys (73). In another study, 36 Dezhou donkeys were divided into two groups and reared on a low-energy (LE) diet and a high-energy (HE) diet. Notably, the average daily feed intake of HE diet (4,262 g/ day) was lower than that of LE diet (4,337 g/ day), but the average daily gain (797 g/ day) was significantly higher than that of LE diet (706 g/day). This indicated that the feed conversion ratio in HE group (0.19) was significantly higher than that in LE group (0.16) (74). Additionally, the addition of yeast polysaccharide to the diet of Dezhou donkeys was shown to increase the average feed intake from 7.96 to 8.09% and the average daily gain from 0.32 kg/d to 0.37 kg/d, yielding positive effects (75).

## 9 Effect of storage and processing conditions on donkey meat quality

Meat aging is a natural process that occurs during storage and involves gradual changes in the contents of moisture, protein, fat, and other substances. One common contributor to the aging of meat is oxidation (76, 77). In one study, at 1, 8, and 15 days after slaughter, the tenderness of donkey meat and its content of PUFA and VOCs were found to change significantly as storage was prolonged (78), consistent with the research reported by Marino et al. (79). Meanwhile, in another study, the effects of different storage periods (1, 6, and 14 days) on the quality of meat from 10 MF donkeys were studied via proteomic methods. The findings indicated that with the increase in the myofibrillar fracture index during the storage process, the shear force, hardness, stickiness, and chewability of donkey meat gradually decrease. These studies collectively show that the quality of donkey meat is susceptible to deterioration during storage due to aging. In order to minimize the adverse effects of aging, it is essential to identify appropriate storage and processing conditions for donkey meat (80). A study examining the effect of low-voltage electrical stimulation on the quality of post-slaughter donkey meat discovered that electrical stimulation can induce a significant pH decline in donkey meat within 6 h of slaughter. Further, it revealed that electrical stimulation during the aging process can significantly reduce the shear force and increase the L* value of donkey meat (81). A volatile oil nanoemulsion containing ε-polylysine was found to preserve the freshness of refrigerated donkey meat by maintaining a stable pH during storage, reducing color changes, and delaying the decline of the elasticity and cohesiveness of refrigerated donkey meat (82). Meanwhile, the dry-curing (cooling, salting, air-drying, fermenting, and aging) of donkey meat was reported to significantly reduce its moisture content while increasing the contents of PUFA and VOCs such as aldehydes and esters (83). Hence, during storage, different methods can be used to process donkey meat, including the use of low-voltage electrical stimulation, which can improve its brightness and tenderness; the addition of a nano-emulsion, which can maintain its freshness; and dry-curing, which can improve its flavor.

## 10 Conclusion

Donkey meat is rich in protein and amino acids and low in fat. After understanding the advantages of donkey meat, consumers can reap the health benefits of this flavorful meat. Meat quality is a collective reflection of the physicochemical characteristics of muscle tissues and is affected by a range of factors. The quality of donkey meat is also determined by various factors, including the characteristics of the animal itself, especially the breed, sex, and age. Understanding the influence of genetic characteristics on donkey meat quality can help producers develop more effective reproductive strategies and select individuals with excellent meat quality genes for breeding, so as to improve the meat quality standard. The sensory properties of meat (tenderness, flavor, etc.) mainly depend on the muscle type and how the meat is stored and processed. With regard to storage and processing, producers can improve processing techniques and use electrical stimulation or dry-curing methods to retain the nutrients and flavor compounds of donkey meat and ensure product quality and taste. It is worth mentioning that the quality of donkey meat can also be affected by the feeding methods and diet used for rearing. Producers can adjust feed formulations and management practices to increase the essential fatty acid content of donkey meat and further improve IMF levels, thereby enhancing meat quality and taste. Notably, gene regulation also plays a key role in determining meat quality, and differences in the molecular profiles and pathways of donkey meat can lead to quality variations. However, because the influence of genetics is extremely complex, more studies are required to fully explore the influence of genetic factors on meat quality. In this review, the factors influencing donkey quality were reviewed in order to provide new ideas for producing high-quality meat. Based on the present review, policymakers can formulate relevant standards and guidelines for donkey meat production and sales and promote the development of the donkey meat industry.
